# Causal associations between hand grip strength and pulmonary function: a two-sample Mendelian randomization study

**DOI:** 10.1186/s12890-023-02720-0

**Published:** 2023-11-21

**Authors:** Xianghu Zhao, Wenyuan Xu, Yanchao Gu, Zhanghua Li, Guiju Sun

**Affiliations:** 1 College of Sports Medicine, Wuhan Sports University, Wuhan, 430079 Hubei Province China; 2 Department of Rehabilitation, Zhongda Hospital, Southeast University, Nanjing, 210009 Jiangsu Province China; 3 Graduate School, Anhui University of Chinese Medicine, Hefei, 230012 Anhui Province China; 4 Department of Orthopedics, Wuhan Third Hospital, Tongren Hospital of Wuhan University, Wuhan, 430074 Hubei Province China; 5 Key Laboratory of Environmental Medicine and Engineering of Ministry of Education, and Department of Nutrition and Food Hygiene, School of Public Health, Southeast University, Nanjing, 210009 Jiangsu Province China

**Keywords:** Hand grip strength, Sarcopenia, Pulmonary function, Causal association, Mendelian randomization

## Abstract

**Background:**

Several observational studies have reported an association between hand grip strength (HGS) and pulmonary function (PF). However, causality is unclear. To investigate whether HGS and PF are causally associated, we performed Mendelian randomization (MR) analyses.

**Methods:**

We identified 110 independent single nucleotide polymorphisms (SNPs) for right-hand grip strength (RHGS) and 103 independent SNPs for left-hand grip strength (LHGS) at the genome-wide significant threshold (*P* < 5 × 10^−8^) from MRC-IEU Consortium and evaluated these related to PF. MR estimates were calculated using the inverse-variance weighted (IVW) method and multiple sensitivity analyses were further performed.

**Results:**

Genetical liability to HGS was positively causally associated with forced vital capacity (FVC) and forced expiratory volume in one second (FEV1), but not with FEV1/FVC. In addition, there was positive causal association between RHGS and FVC (OR=1.519; 95% CI, 1.418-1.627; *P*=8.96E-33), and FEV1 (OR=1.486; 95% CI, 1.390-1.589; *P*=3.19E-31); and positive causal association between LHGS and FVC (OR=1.464; 95% CI, 1.385-1.548; *P*=2.83E-41) and FEV1 (OR=1.419; 95% CI, 1.340-1.502; *P*=3.19E-33). Nevertheless, no associations were observed between RHGS and FEV1/FVC (OR=0.998; 95% CI, 0.902-1.103; *P*=9.62E-01) and between LHGS and FEV1/FVC (OR=0.966; 95% CI, 0.861-1.083; *P*=5.52E-01). Similar results were shown in several sensitivity analyses.

**Conclusion:**

Our study provides support at the genetic level that HGS is positively causally associated with FVC and FEV1, but not with FEV1/FVC. Interventions for HGS in PF impairment deserve further exploration as potential indicators of PF assessment.

**Supplementary Information:**

The online version contains supplementary material available at 10.1186/s12890-023-02720-0.

## Introduction

Sarcopenia reduces the quality of life of older people and is a high-risk factor for complications [1] such as falls, fractures, dysphagia, respiratory dysfunction, and cardiovascular disease [2, 3]. Sarcopenia is intrinsic to the concept of frailty and represents a special target group for frailty prevention [4]. The term “sarcopenia” was coined in 1989 to describe the progressive age-related loss of muscle mass [5]. More recently, the Asian Working Group for Sarcopenia 2019 (AWGS2019) in the elderly changed the diagnostic algorithm to focus on muscle strength and recommended early detection and treatment for sarcopenia [6]. Expensive and time-consuming radiological evaluation methods (such as CT, MRI, and dual-energy X-ray absorptiometry) are used in clinical practice to measure body composition (including total lean body mass and appendicular lean body mass) [7]. It has been shown that in patients with sarcopenia, the quadriceps muscles are the first to atrophy. Therefore, the use of ultrasound to measure the quadriceps mass as a new diagnostic method to improve the assessment and management of sarcopenia has been proposed by the International Society of Physical and Rehabilitation Medicine (ISPRM) [8]. Recently, hand grip strength (HGS) has become a convenient measurement for assessing overall muscle strength that is simple, fast, and standardized. Previous studies have reported a strong correlation between HGS and muscle mass, nutritional status, and walking performance [9, 10]. Moreover, HGS is believed a crucial index when diagnosing sarcopenia as weak HGS is a significant predictor of low muscle mass and a characteristic of decreased physical function [11, 12].

Impaired pulmonary function (PF) is associated not only with respiratory complications for instance pneumonia and bronchitis, but also with all-cause mortality and cardiovascular disease [13, 14]. Thus, the early detection of older people at high risk of impaired PF, from a public health perspective, is of importance. A large body of emerging epidemiological research has found an association between HGS and a variety of detrimental health outcomes in older people. Meanwhile, Leong et al. found that grip strength was inversely associated with myocardial infarction, all-cause mortality, non-cardiovascular mortality, cardiovascular mortality, and stroke [15]. Age-related decline in skeletal muscles also consists of a loss of respiratory muscle mass and strength and thus may contribute to impaired PF [16]. The relations HGS and PF have been investigated, with most studies paid more attention to individuals in nursing home settings or hospitals or using only a few participants [17, 18]. It has also been known about community-dwelling women aged 65 and older population in the Korean National Health and Nutrition Examination Survey (KNHANES) [10]. However, most of the evidence for the relations comes from observational studies, which are inconclusive in identifying the causality because of the possibility of residual confounding and reverse causation.

For causality, Mendelian randomization (MR) is an increasingly applied analysis method that can employ genetic variations from recent genome-wide association studies (GWAS) as instrumental variables (IVs) to clarify the causal relationship between exposure and outcomes, and decrease potential confounding factors in observational studies [19]. Therefore, the aim of this study was to perform the two-sample MR analyses to examine the potential causality between HGS and PF, including Forced Expiratory Volume in one second (liters; FEV1), Forced Vital Capacity (liters; FVC), and Forced Expiratory Volume in one second/ Forced Vital Capacity ratio (percentage; FEV1/ FVC ratio). And multiple complementary analyses also have been conducted to test the robustness of the results.

## Methods

### Study design

**Fig. 1:**
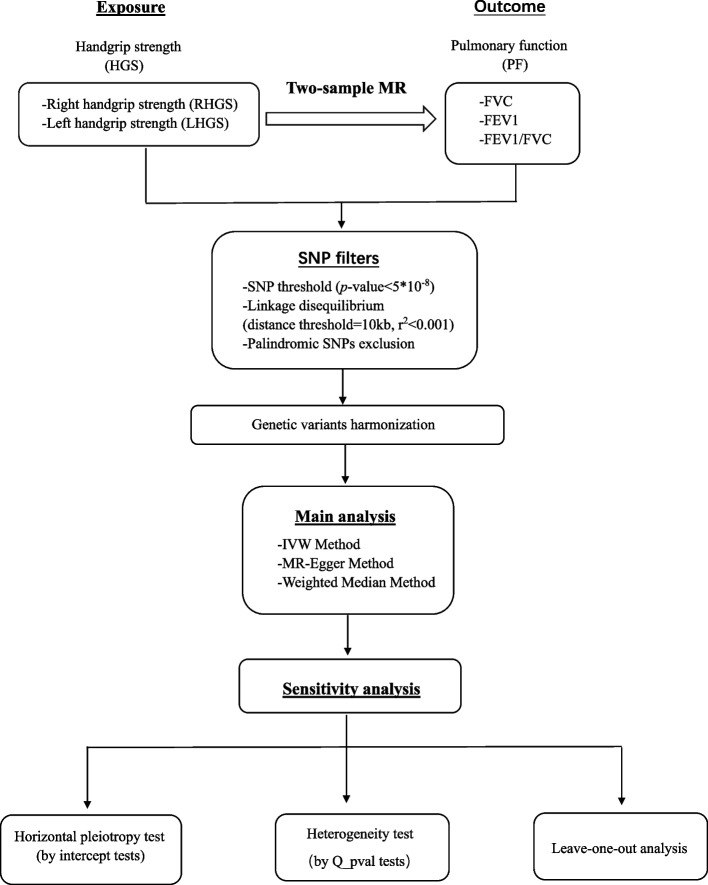
The steps of Mendelian randomization (MR) analyses

**Fig. 2:**
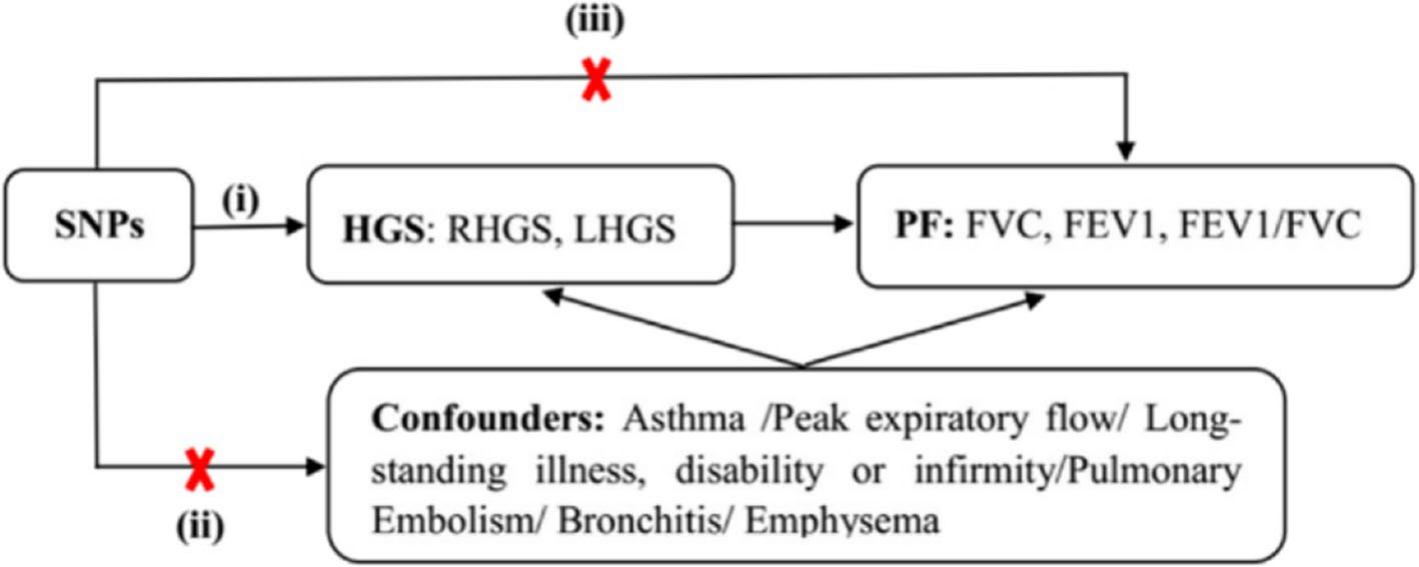
Three key assumptions of the MR study. (i) SNPs are robustly associated with HGS (hand grip strength); (ii) SNPs are independent of other known confounders; (iii) SNPs affect the risk of PF (pulmonary function) only through HGS. The red X means that the SNPs selected as the instrumental variables are not associated with the confounders and the outcomes directly.

The two-sample MR analyses flow chart is shown in Fig. 1. In short, the genetic variations used as IVs must follow three key assumptions: (i) the genetic variants are strongly associated with HGS (each genetic variant for HGS reached GWAS [*P* < 5 × 10^−8^], and the threshold of F-statistic); (ii), the genetic variants should not be associated with any confounders; (iii) the genetic variants effect the outcome only via the HGS (no horizontal pleiotropy) (Fig. 2). All summary statistics presented in this study were derived from published GWAS (https://gwas.mrcieu.ac.uk/) on HGS and PF (Supplementary Table 1).

### Data sources for HGS and selection of IVs

Grip strength was measured using a hand-held dynamometer and multiple measurements were taken to obtain the maximum value. Exclusion criteria for grip strength analysis included age <65 years, non-Caucasian origin via self-report or 1 clustering of the GWAS data, missing grip strength data, self-reported pain, surgery, or osteoarthritis in the dominant hand was considered [20]. The summary statistic for right-handgrip strength (RHGS) and left-handgrip strength (LHGS) were derived from a recently released GWAS of the MRC-IEU Consortium, which included 461,089 participants and 461,026 participants from Europe [21]. In Brief, this GWAS examined two HGS phenotypes including RHGS (n = 9,851,867) and LHGS (n = 9,851,867). In the MRC-IEU Consortium, we adopted absolute rather than relative HGS as a marker because of absolute HGS may more associated with physical capability than relative HGS [22].

To meet the first assumption of MR analyses, this study selected 110 independent single-nucleotide polymorphisms (SNPs) associated with “RHGS” and 103 independent SNPs associated with “LHGS” at a genome-wide significance level (*P* < 5×10^−8^), using the PLINK clumping algorithm (R^2^ > 0.001, Kb = 10K) from the GWAS mentioned above. F-statistics were generated to assess the strength of selected SNPs using the following formula: $\mathrm{F}=\frac{{\mathrm{R}}^{2}(\mathrm{N}-2)}{(1-{\mathrm{R}}^{2})}$. Where, R^2^ is the percentage of the variability in HGS explained by the selected SNPs and N represents the sample size of the GWAS [23]. An F-statistic<10 indicates a low risk of weak instrument bias in MR analyses [23].

### Data sources for pulmonary function

We derived three genetic instruments for PF: FVC, FEV1, and FEV1/ FVC. GWAS summary statistics for PF were extracted from: the MRC-IEU consortium for FVC and FEV1; the NA Consortium for FEV1/ FVC. Details of the datasets included in the analyses were shown in Supplementary Table S1.

### Statistical analyses

We ran a two-sample MR method using summary data from the MRC-IEU Consortium Genome-Wide Association Studies (GWAS). After extraction of statistic and harmonization of the effect alleles by GWAS, the MR estimates of the effect of HGS on PF was calculated using the Wald estimates. The Delta method was used to account for possible measurement errors in the estimation of the causality between HGS and PF [24, 25]. The fixed-effects inverse variance weighted (IVW) method was applied to evaluate the final effect estimate. Scatter plots of the MR effects estimated by each method were also provided.

In the IVW analyses, pleiotropy of SNPs may affect causal estimates and bias the results. In this study, we calculated the Cochran’s Q to test the heterogeneity caused by different SNPs in the fixed-effects IVW. Cochran’s Q *P*-value < 0.05 indicated the presence of heterogeneity, and of horizontal pleiotropy [26]. In the case of potential horizontal pleiotropy, the random-effects IVW method would be used. MR-Egger intercept test was performed to identify potential directional pleiotropy, with an intercept *P*-value < 0.05 indicating significant pleiotropic bias [27].

In addition, we also conducted several sensitivity analyses to further ensure the robustness of our results, including the MR-Egger regression method [27], simple mode, weighted median method, and leave-one-SNP-out method. To rule out the IVs related to any confounders that may affect HGS and PF, we also searched each selected SNP and its proxies in Phenoscanner (http://www.phenoscanner.medschl.cam.ac.uk/) [28] for previously detected associations (*P*-value < 5 × 10^−8^) with relevant confounders or PF. In this study, asthma, peak expiratory flow, bronchitis, pulmonary embolism, long-standing illness, and disability or infirmity were regarded as confounders. We repeated the two-sample MR analyses mentioned above after removing the SNPs related to relevant confounders or PF.

A two-sided *P*-value<0.05 was set as suggestive significance, and due to the multiple comparisons, we further adopted a Bonferroni corrected threshold for statistical significance *P*-value< 0.008 (0.05/6). All MR analyses were conducted using R software (version 4.2.1; www.r-project.org) with the R packages “Mendelian Randomization” and “Two-sample MR”.

## Results

### SNP Selection and validation

In general, we included studies published between 2018 and 2019 based mainly on European population (Supplementary Table S1). Independent SNPs included for analyses as IVs are shown in Supplementary Tables S2 and S3. They show the characteristics of all correlated SNPs for HGS. Overall, we extracted 110 and 103 independent SNPs that reached genome-wide significance from RHGS and LHGS, respectively. Among all selected SNPs, the F-statistics were higher than 10 and ranged from 30 to 192. In the Phenoscanner, we detected 120 selected SNPs that were considered to be related to confounders or PF for HGS, respectively. (Supplementary Table S4, S5)

### RHGS and PF

The IVW analyses indicated that the genetically predicted RHGS per standard deviation (SD) increase was positive associated with FVC (OR=1.519; 95% CI, 1.418-1.627; *P*=8.96E-33), and FEV1 (OR=1.486; 95% CI, 1.390-1.589; *P*=3.19E-31). On the contrary, no association was observed for FEV1/FVC (OR=0.998; 95% CI, 0.902-1.103; *P*=9.62E-01) (Table 1). For FVC, FEV1, and FEV1/FVC, the weighted-median and MR-Egger analyses indicated consistent estimates (Table1). No evidence of directional pleiotropy was identified. The heterogeneity was higher for indicators of PF. Therefore, an IVW analysis under a random-effects model was applied to mitigate the influence of heterogeneity (Table 2).

**Table 1:** Mendelian randomization estimates between right-hand grip strength and pulmonary function

**Outcomes**	**SNP selection**	**No. of SNPs**	**IVW**		**MR Egger**		Weighted median		Simple mode		Weighted mode	
			**OR (95%CI)**	***P-value***	**OR (95%CI)**	***P-value***	**OR (95%CI)**	***P-value***	**OR (95%CI)**	***P-value***	**OR (95%CI)**	***P-value***
FVC	All	169	1.987	2.31E-60	1.742	2.71E-04	1.622	4.42E-63	1.760	4.43E-14	1.697	1.98E-10
			(1.830-2.157)		(1.300-2.333)		(1.533-1.717)		(1.539-2.013)		(1.457-1.978)	
	Remove	104	1.519	8.96E-33	1.410	8.86E-03	1.480	5.61E-35	1.729	1.03E-08	1.692	1.43E-02
			(1.418-1.627)		(1.095-1.814)		(1.390-1.575)		(1.456-2.055)		(1.119-2.559)	
FEV1	All	169	1.839	2.03E-58	1.411	1.06E-02	1.562	1.28E-50	1.641	1.75E-06	1.504	5.19E-06
			(1.707-1.980)		(1.087-1.831)		(1.473-1.656)		1.349-1.996)		(1.269-1.782)	
	Remove	104	1.486	3.19E-31	1.266	6.05E-0	1.379	5.69E-22	1.690	5.30E-06	1.352	3.64E-02
			(1.390-1.589)		(0.992-1.615)		(1.292-1.472)		(1.365-2.095)		(1.023-1.786)	
FEV1/FVC	All	165	0.938	2.60E-01	0.670	4.90E-02	0.985	6.58E-01	0.990	9.31E-01	1.003	9.78E-01
			(0.839-1.049)		(0.451-0.995)		(0.919-1.055)		(0.792-1.237)		(0.820-1.226)	
			0.998		0.852		1.048		1.025		1.035	
	Remove	101	(0.902-1.103)	9.62E-01	(0.589-1.232)	3.96E-01	(0.962-1.142)	2.85E-01	(0.801-1.312)	8.46E-01	(0.848-1.263)	7.36E-01

**Table 2:** Tests of pleiotropy of selected SNPs and heterogeneity between SNPs. (RHGS)

**Outcomes**	**Pleiotropy Test**			**Heterogeneity Test**	
	**Intercept**	**Beta (SE)**	***P*-Value**	**Cochran’s Q**	***P*-Value**
FVC	0.001	0.001	0.548	465.73	1.50E-47
FEV1	0.002	0.001	0.183	394.42	1.09E-35
FEV1/FVC	0.002	0.002	0.385	443.34	9.84E-45

Scatter plot and forest plot of the association between RHGS and PF are shown in Supplementary Figure S1 and Supplementary Figure S2, respectively, where similar results can be observed. The leave-one-out sensitivity analyses, as shown in Supplementary Figure S3, indicated that the overall estimates were not disproportionately influenced by any individual SNP. The funnel plot in Supplementary Figure S4 also revealed no evidence of horizontal pleiotropy.

### LHGS and PF

The IVW analyses showed that genetically predicted LHGS per standard deviation (SD) increase was positive related to FVC (OR =1.464; 95% CI, 1.385-1.548; *P*=2.83E-41), and FEV1 (OR=1.419; 95% CI, 1.340-1.502; *P*=3.19E-33). Conversely, no association was observed for FEV1/FVC (OR=0.966; 95% CI, 0.861-1.083; *P*=5.52E-01) (Table 3). For FVC, FEV1, and FEV1/FVC, the weighted-median and MR-Egger analyses indicated consistent estimates (Table 3). No evidence of directional pleiotropy was detected. The heterogeneity was higher for indicators of PF. Hence, an IVW analysis under a random-effects model was applied to mitigate the influence of heterogeneity (Table 4).

**Table 3:** Mendelian randomization estimates between left-hand grip strength and pulmonary function

**Outcomes**	**SNP selection**	**No. of SNPs**	**IVW**		**MR Egger**		Weighted median		Simple mode		Weighted mode	
			**OR (95%CI)**	***P-value***	**OR (95%CI)**	***P-value***	**OR (95%CI)**	***P-value***	**OR (95%CI)**	***P-value***	**OR (95%CI)**	***P-value***
FVC	All	155	1.907	1.05E-62	2.257	3.74E-07	1.604	1.05E-62	1.633	7.05E-11	1.622	1.05E-08
			(1.759-2.068)		(1.672-3.048)		(1.518-1.695)		(1.424-1.873)		(1.387-1.897)	
	Remove	103	1.464	2.83E-41	1.239	4.33E-02	1.501	4.35E-41	1.622	1.84E-09	1.629	8.07E-07
			(1.385-1.548)		(1.009-1.521)		(1.414-1.592)		(1.405-1.872)		(1.358-1.953)	
FEV1	All	155	1.773	1.52E-49	1.875	2.31E-05	1.534	9.77E-49	1.591	6.40E-07	1.557	9.10E-06
			(1.643-1.912)		(1.414-2.486)		(1.449-1.624)		(1.335-1.896)		(1.289-1.881)	
	Remove	103	1.486	3.19E-33	1.235	5.36E-02	1.419	7.03E-30	1.586	1.57E-06	1.586	2.60E-03
			(1.340-1.502)		(0.999-1.527)		(1.336-1.507)		(1.328-1.893)		(1.183-2.125)	
FEV1/FVC	All	152	0.938	2.66E-01	0.796	3.17E-01	0.978	5.48E-01	0.913	2.66E-01	0.966	7.14E-01
			(0.830-1.053)		(0.150-1.242)		(0.910-1.051)		(0.746-1.117)		(0.801-1.164)	
			0.998		1.254		0.984		0.840		0.921	
	Remove	101	(0.861-1.083)	5.52E-01	(0.815-1.930)	3.06E-01	(0.901-1.075)	7.27E-01	(0.652-1.083)	1.81E-01	(0.722-1.175)	5.11E-01

**Table 4:** Tests of pleiotropy of selected SNPs and heterogeneity between SNPs. (LHGS)

**Outcomes**	**Pleiotropy Test**			**Heterogeneity Test**	
	**Intercept**	**Beta (SE)**	***P*-Value**	**Cochran’s Q**	***P*-Value**
FVC	0.002	0.001	0.101	312.79	2.97E-23
FEV1	0.002	0.001	0.186	295.71	1.80E-41
FEV1/FVC	-0.003	0.003	0.220	605.84	2.09E-73

Scatter plot, forest plot, the results of the leave-one-out sensitivity analyses, and the funnel plot of the association between LHGS and PF are shown in Supplementary Figure S1, Supplementary Figure S2, Supplementary Figure S3, and Supplementary Figure S4, respectively, where similar results can be observed.

## Discussion

In this study, we explored the causal associations between HGS and PF by using two-sample MR analyses. We confirmed that greater HGS was significantly causally associated with the high-quality PF. In addition, there was a significant association between both right- and left- HGS and FVC, FEV1. Besides, no significant association was found between HGS and FEV1/FVC.

The observational studies that revealed HGS may be associated with PF and have aroused the interests of researchers to search for more evidence to demonstrate the causal association [29]. In this MR study, our results are consistent with those of previous observational studies that have found that HGS as a measurement of sarcopenia may suggest a decline in PF in older people [17, 18]. Positive associations were found between HGS and maximal inspiratory pressure (MIP) and maximal expiratory pressure (MEP) in bivariate correlation analyses of 62 Turkish nursing home residents with a mean age of 70.5, but only MIP was significantly related to HGS in the multiple linear regression analyses [17]. Recently, the association between HGS and PF was researched in 50 individuals older than 70 in an acute medical ward [18]. Of spirometry measures including peak expiratory flow, FEV1, FVC, and peak cough flow, only peak cough flow was associated with HGS [18]. It remains controversial why the causal association between HGS and FVC, FEV1, and FEV1/FVC. Our results were different from that in the previous studies, but several explanations can be given. The previous observational studies have been performed in nursing homes, hospitals, and community-dwelling older people and have had relatively small sample sizes. Additionally, the important parameters associated with PF, such as asthma, peak expiratory flow, long-standing illness, disability or infirmity, pulmonary embolism, bronchitis, and emphysema, have not been adequately adjusted for in most previous studies. Adjusting for potential confounding variables helps to clarify the true causal association between HGS and PF from a SNPs perspective.

To our knowledge, we believe this is the first MR study to document a positive causal association of HGS with FVC and FEV1 and no causal association with FEV1/FVC, and that the association found between them was from a genetic level using MR analyses. Some mechanisms could explain the significant relationships between HGS and PF. Skeletal muscle mass decreases with age and ultimately results in the loss of respiratory muscle mass and strength, for instance in the diaphragm muscle [30]. Respiratory muscle strength plays a crucial role in the respiratory network, which adjusts the cross talk between PF and the respiratory muscles to maintain adequate ventilation [31]. The activated respiratory muscles developed a pressure gradient in the intrathoracic, and air is exchanged over the alveolar surface. It has been reported that major parameters that represent respiratory muscle strength such as MEP and MIP are related to peripheral muscle strength, which shows that peripheral muscle strength and respiratory muscle strength are interrelated [32]. In another recent study, HGS as a measurement of peripheral muscle strength had a significant positive correlation with MEP and MIP [33]. HGS is also closely related to PF in chronic obstructive pulmonary disease (COPD) patients. Qaisar et al. elucidated that the expression of CC16 and STA in serum showed a positive correlation with FEV1 and HGS in COPD patients [34], while Kyomoto et al. found that HGS correlates more strongly with 6-min walk test distance (6-MWD) than other factors, and could be used as one of the predictors of exercise capacity in COPD patients [35]. Additionally, Samarghandi et al. showed that HGS and peak inspiratory flow rate (PIFR) in acute exacerbation of COPD (AECOPD) hospitalized patients have a positive correlation and can be used as one of the predictors of inspiratory muscle strength [36]. Besides, it has been revealed that weak respiratory muscle strength occurs at the beginning of a causal chain that can contribute to poor PF, as well as leading to death [37].

A decline in respiratory muscle strength may affect FVC and FEV1 more than FEV1/FVC as the latter typically depends not only upon adequate respiratory muscle strength, but also on airway status. In this respect, the association between HGS and FVC and FEV1 was more prominent than the association with FEV1/FVC in the present study. Moreover, the positive causal association between HGS and resistance training and physical activity indicates that people who exercise regularly may have greater capacity to improve PF and skeletal muscle power.

Our study has several evident strengths. Firstly, this was the first two-sample MR study to evaluate the causal associations of HGS with FVC, FEV1, and FEV1/FVC by using the recently published GWAS. Secondly, various complementary analyses were adopted to address pleiotropic bias and confirm the robustness of our results. Thirdly, we repeated the analyses after excluding the IVs related to any confounders or PF and the result was consistent.

Additionally, several potential limitations were also worth acknowledging. To begin with, while no apparent pleiotropy was identified for the IVs used, the possibility of residual pleiotropy still cannot be completely ruled out. There may be other undiscovered causal pathways of HGS with PF. Next, SNPs associated with HGS were applied from the GWAS of MRC-IEU Consortium, which includes participants aged between 40 to 70 years from Europe. Furthermore, we do not have the demographic information which restricts the generalizability of our results. Thus, further studies are warranted to confirm our findings on other populations. Then, though HGS is an objective and common marker of muscular strength, it mainly represents upper body strength. Finally, because the causal relationship was evaluated using MR method depended on the genetic information of each trait, the result should be interpreted with caution [38], with the understanding that the development of HGS and PF were multifactorial and involved interactions among plenty of psycho-social-environmental factors [39]. However, this bias would likely be minimal on account of the limited overlap in the samples between HGS and PF. In future studies, we will conduct prospective cohort studies to provide even stronger evidence of this causal relationship.

## Conclusion

In summary, our study provides genetic evidence supporting a causal relationship between HGS on FVC and FEV1, but not FEV1/FVC. Given the health implications of PF, timely detection of lower HGS in older adults may be useful in assessment of potential PF impairment. Additionally, in clinical interventions for patients with sarcopenia, it is important to focus not only on interventions targeting appendicular muscles but also on core muscle groups, particularly respiratory muscle groups.

### Supplementary Information

**Additional file 1:** **Supplementary Figure S1.** Scatter plot of the association of hand grip strength with pulmonary function. **Supplementary Figure S2.** Forest plot of the association of hand grip strength with pulmonary function. **Supplementary Figure S3.** Leave-one-out sensitivity analysis of the association of hand grip strength with pulmonary function. **Supplementary Figure S4.** Funnel plot of the association of hand grip strength with pulmonary function. **Supplementary Table S1.** Baseline characteristics of hand grip strength and pulmonary function. **Supplementary Table S2.** Single nucleotide polymorphisms used as instrumental variables in the Mendelian randomization analysis of right-hand grip strength. **Supplementary Table S3.** Single nucleotide polymorphisms used as instrumental variables in the Mendelian randomization analysis of left-hand grip strength. **Supplementary Table S4.** SNPs of RHGS excluded from Mendelian randomization analysis. **Supplementary Table S5.** SNPs of LHGS excluded from Mendelian randomization analysis. STROBE-MR checklist of recommended items to address in reports of Mendelian randomization studies.

## Data Availability

The GWAS data of hand grip strength was retrieved from IEU-OpenGWAS project (https://gwas.mrcieu.ac.uk/datasets/ukb-b-10215/, https://gwas.mrcieu.ac.uk/datasets/ukb-b-7478/) online platform. The GWAS data of pulmonary function were retrieved from IEU-OpenGWAS project (https://gwas.mrcieu.ac.uk/datasets/ukb-b-7953/, https://gwas.mrcieu.ac.uk/datasets/ukb-b-19657/, https://gwas.mrcieu.ac.uk/datasets/ebi-a-GCST007431/).

## Abbreviations

HGS: Hand grip strength
PF: Pulmonary function
MR: Mendelian randomization
SNPs: Single nucleotide polymorphisms
RHGS: Right-hand grip strength
LHGS: Left-hand grip strength
IVW: Inverse-variance weighted
FVC: Forced vital capacity
FEV1: Forced expiratory volume in one second
AWGS2019: Asian Working Group for Sarcopenia 2019
KNHANES: Korean National Health and Nutrition Examination Survey
GWAS: Genome-wide association studies
IVs: Instrumental variables
MIP: Maximal inspiratory pressure
MEP: Maximal expiratory pressure
COPD: Chronic obstructive pulmonary disease
6-MWD: 6-min walk test distance
PIFR: Peak inspiratory flow rate
AECOPD: Acute exacerbation of COPD
