# Supplementary material for: Causal associations between hand grip strength and pulmonary function: a two-sample Mendelian randomization study
Source: BMC Pulm Med. 2023 Nov 21;23:459. doi: 10.1186/s12890-023-02720-0 (PMC10664596; doi:10.1186/s12890-023-02720-0)
Supplement: Supplementary file 1 — Additional file 1: Supplementary Figure S1. Scatter plot of the association of hand grip strength with pulmonary function. Supplementary Figure S2. Forest plot of the association of hand grip strength with pulmonary function. Supplementary Figure S3. Leave-one-out sensitivity analysis of the association of hand grip strength with pulmonary function. Supplementary Figure S4. Funnel plot of the association of hand grip strength with pulmonary function. Supplementary Table S1. Baseline characteristics of hand grip strength and pulmonary function. Supplementary Table S2. Single nucleotide polymorphisms used as instrumental variables in the Mendelian randomization analysis of right-hand grip strength. Supplementary Table S3. Single nucleotide polymorphisms used as instrumental variables in the Mendelian randomization analysis of left-hand grip strength. Supplementary Table S4. SNPs of RHGS excluded from Mendelian randomization analysis. Supplementary Table S5. SNPs of LHGS excluded from Mendelian randomization analysis. STROBE-MR checklist of recommended items to address in reports of Mendelian randomization studies. [file 12890_2023_2720_MOESM1_ESM.zip › sumplmentary/STROBE-MR-checklist.pdf]

STROBE-MR checklist of recommended items to address in reports of Mendelian randomization studies[1 2]

Item No.	Section		Checklist item	Page No.	Relevant text from manuscript
1	**TITLE and ABSTRACT**		Indicate Mendelian randomization (MR) as the study's design in the title and/or the abstract if that is a main purpose of the study	1	Lines 1-2 & 23-25
	**INTRODUCTION**				
2	**Background**		Explain the scientific background and rationale for the reported study. What is the exposure? Is a potential causal relationship between exposure and outcome plausible? Justify why MR is a helpful method to address the study question	3	Lines 74-81
3	**Objectives**		State specific objectives clearly, including pre-specified causal hypotheses (if any). State that MR is a method that, under specific assumptions, intends to estimate causal effects	3	Lines 85-90
	**METHODS**				
4	**Study design and data sources**		Present key elements of the study design early in the article. Consider including a table listing sources of data for all phases of the study. For each data source contributing to the analysis, describe the following:		
		a)	Setting: Describe the study design and the underlying population, if possible. Describe the setting, locations, and relevant dates, including periods of recruitment, exposure, follow-up, and data collection, when available.	4-5	Lines 145-150
		b)	Participants: Give the eligibility criteria, and the sources and methods of selection of participants. Report the sample size, and whether any power or sample size calculations were carried out prior to the main analysis	4-5	Lines 145-150
		c)	Describe measurement, quality control and selection of genetic variants	4	Figure 1
		d)	For each exposure, outcome, and other relevant variables, describe methods of assessment and diagnostic criteria for diseases	4	Lines 141-144
		e)	Provide details of ethics committee approval and participant informed consent, if relevant	13	Lines 368-369
5	**Assumptions**		Explicitly state the three core IV assumptions for the main analysis (relevance, independence and exclusion restriction) as well assumptions for any additional or sensitivity analysis	3-4	Lines 95-98
6	**Statistical methods: main analysis**		Describe statistical methods and statistics used		

		a) Describe how quantitative variables were handled in the analyses (i.e., scale, units, model)	5	Lines 151-158
		b) Describe how genetic variants were handled in the analyses and, if applicable, how their weights were selected	5	Lines 151-158
		c) Describe the MR estimator (e.g. two-stage least squares, Wald ratio) and related statistics. Detail the included covariates and, in case of two-sample MR, whether the same covariate set was used for adjustment in the two samples	5	Lines 151-158
		d) Explain how missing data were addressed	6	Lines 181-186
		e) If applicable, indicate how multiple testing was addressed	6	Lines 179-181
7	**Assessment of assumptions**	Describe any methods or prior knowledge used to assess the assumptions or justify their validity	6	Lines 179-181
8	**Sensitivity analyses and additional analyses**	Describe any sensitivity analyses or additional analyses performed (e.g. comparison of effect estimates from different approaches, independent replication, bias analytic techniques, validation of instruments, simulations)	5-6	Lines 172-181
9	**Software and pre-registration**			
		a) Name statistical software and package(s), including version and settings used	6	Lines 190-191
		b) State whether the study protocol and details were pre-registered (as well as when and where)	None	
	**RESULTS**			
10	**Descriptive data**			
		a) Report the numbers of individuals at each stage of included studies and reasons for exclusion. Consider use of a flow diagram	4	Figure 1
		b) Report summary statistics for phenotypic exposure(s), outcome(s), and other relevant variables (e.g. means, SDs, proportions)		Supplementary Tables
		c) If the data sources include meta-analyses of previous studies, provide the assessments of heterogeneity across these studies	None	
		d) For two-sample MR:<br>i. Provide justification of the similarity of the genetic variant-exposure associations between the exposure and outcome samples	10	Lines 266-267

		ii. Provide information on the number of individuals who overlap between the exposure and outcome studies	6	Lines 194-202
11	**Main results**			
		a) Report the associations between genetic variant and exposure, and between genetic variant and outcome, preferably on an interpretable scale	6-7	Lines 204-207 & 219-222
		b) Report MR estimates of the relationship between exposure and outcome, and the measures of uncertainty from the MR analysis, on an interpretable scale, such as odds ratio or relative risk per SD difference	6-7	Lines 204-207 & 219-222
		c) If relevant, consider translating estimates of relative risk into absolute risk for a meaningful time period	6-7	Lines 204-207 & 219-222
		d) Consider plots to visualize results (e.g. forest plot, scatterplot of associations between genetic variants and outcome versus between genetic variants and exposure)		Supplementary Figures S1 & S2
12	**Assessment of assumptions**			
		a) Report the assessment of the validity of the assumptions	6-7	Lines 207-211 & 222-226
		b) Report any additional statistics (e.g., assessments of heterogeneity across genetic variants, such as I^2, Q statistic or E-value)	6-7	Lines 207-211& 222-226
13	**Sensitivity analyses and additional analyses**			
		a) Report any sensitivity analyses to assess the robustness of the main results to violations of the assumptions	6-7	Lines 209-211 & 224-226
		b) Report results from other sensitivity analyses or additional analyses	6-7	Lines 209-211 & 224-226
		c) Report any assessment of direction of causal relationship (e.g., bidirectional MR)	6-7	Lines 209-211 & 224-226
		d) When relevant, report and compare with estimates from non-MR analyses	6-7	Lines 209-211 & 224-226
		e) Consider additional plots to visualize results (e.g., leave-one-out analyses)	7	Lines 212-217 & 227-230
	**DISCUSSION**			
14	**Key results**	Summarize key results with reference to study objectives	10	Lines 261-265
15	**Limitations**	Discuss limitations of the study, taking into account the validity of the IV assumptions, other sources of potential bias, and imprecision. Discuss both direction and magnitude of any potential bias and any efforts to address them	12	Lines 320-334

16	**Interpretation**			
		a) Meaning: Give a cautious overall interpretation of results in the context of their limitations and in comparison with other studies	10-12	Lines 226-314
		b) Mechanism: Discuss underlying biological mechanisms that could drive a potential causal relationship between the investigated exposure and the outcome, and whether the gene-environment equivalence assumption is reasonable. Use causal language carefully, clarifying that IV estimates may provide causal effects only under certain assumptions	10-12	Lines 226-314
		c) Clinical relevance: Discuss whether the results have clinical or public policy relevance, and to what extent they inform effect sizes of possible interventions	12	Lines 337-342
17	**Generalizability**	Discuss the generalizability of the study results (a) to other populations, (b) across other exposure periods/timings, and (c) across other levels of exposure	12	Lines 337-342
	**OTHER INFORMATION**			
18	**Funding**	Describe sources of funding and the role of funders in the present study and, if applicable, sources of funding for the databases and original study or studies on which the present study is based	13	Lines 384-389
19	**Data and data sharing**	Provide the data used to perform all analyses or report where and how the data can be accessed, and reference these sources in the article. Provide the statistical code needed to reproduce the results in the article, or report whether the code is publicly accessible and if so, where	13	Lines 374-378
20	**Conflicts of Interest**	All authors should declare all potential conflicts of interest	13	Lines 380-382

This checklist is copyrighted by the Equator Network under the Creative Commons Attribution 3.0 Unported (CC BY 3.0) license.

1. Skrivankova VW, Richmond RC, Woolf BAR, Yarmolinsky J, Davies NM, Swanson SA, et al. Strengthening the Reporting of Observational Studies in Epidemiology using Mendelian Randomization (STROBE-MR) Statement. JAMA. 2021;under review.

2. Skrivankova VW, Richmond RC, Woolf BAR, Davies NM, Swanson SA, VanderWeele TJ, et al. Strengthening the Reporting of Observational Studies in Epidemiology using Mendelian Randomisation (STROBE-MR): Explanation and Elaboration. BMJ. 2021;375:n2233.
