# Supplementary material for: Causal associations between hand grip strength and pulmonary function: a two-sample Mendelian randomization study
Source: BMC Pulm Med. 2023 Nov 21;23:459. doi: 10.1186/s12890-023-02720-0 (PMC10664596; doi:10.1186/s12890-023-02720-0)
Supplement: Supplementary file 1 — Additional file 1: Supplementary Figure S1. Scatter plot of the association of hand grip strength with pulmonary function. Supplementary Figure S2. Forest plot of the association of hand grip strength with pulmonary function. Supplementary Figure S3. Leave-one-out sensitivity analysis of the association of hand grip strength with pulmonary function. Supplementary Figure S4. Funnel plot of the association of hand grip strength with pulmonary function. Supplementary Table S1. Baseline characteristics of hand grip strength and pulmonary function. Supplementary Table S2. Single nucleotide polymorphisms used as instrumental variables in the Mendelian randomization analysis of right-hand grip strength. Supplementary Table S3. Single nucleotide polymorphisms used as instrumental variables in the Mendelian randomization analysis of left-hand grip strength. Supplementary Table S4. SNPs of RHGS excluded from Mendelian randomization analysis. Supplementary Table S5. SNPs of LHGS excluded from Mendelian randomization analysis. STROBE-MR checklist of recommended items to address in reports of Mendelian randomization studies. [file 12890_2023_2720_MOESM1_ESM.zip › sumplmentary/Supplementary Figure Captions.pdf]

Supplementary Figure Captions

Supplementary Figure S1. Scatter plot of the association of hand grip strength with pulmonary function.

A: right-hand grip strength and FVC; B: right-hand grip strength and FEV1; C: right-hand grip strength and FEV1/FVC; D: left-hand grip strength and FVC; E: left-hand grip strength and FEV1; F: left-hand grip strength and FEV1/FVC;

Each black dot indicates a SNP, plotted by the estimate of SNP on individual hand grip strength and the estimate of SNP on the risk of pulmonary function with standard error bars. The slopes of the lines correspond to causal estimates using each of the different methods.

SNP: single nucleotide polymorphism

Supplementary Figure S2. Forest plot of the association of hand grip strength with pulmonary function.

A: right-hand grip strength and FVC; B: right-hand grip strength and FEV1;

C: right-hand grip strength and FEV1/FVC; D: left-hand grip strength and FVC; E: left-hand grip strength and FEV1; F: left-hand grip strength and FEV1/FVC;

The dot and bar indicate the causal estimate of individual hand grip strength on risks of pulmonary function.

Supplementary Figure S3. Leave-one-out sensitivity analysis of the association of hand grip strength with pulmonary function.

A: right-hand grip strength and FVC; B: right-hand grip strength and FEV1; C: right-hand grip strength and FEV1/FVC; D: left-hand grip strength and FVC; E: left-hand grip strength and FEV1; F: left-hand grip strength and FEV1/FVC;

The dot and bar indicate the estimates and 95% confidence interval when the specific single nucleotide polymorphism is removed.

Supplementary Figure S4. Funnel plot of the association of hand grip strength with pulmonary function.

A: right-hand grip strength and FVC; B: right-hand grip strength and FEV1; C: right-hand grip strength and FEV1/FVC; D: left-hand grip strength and FVC; E: left-hand grip strength and FEV1; F: left-hand grip strength and FEV1/FVC;

Each black dot indicates a single nucleotide polymorphism.
