# Supplementary material for: Causal associations between hand grip strength and pulmonary function: a two-sample Mendelian randomization study
Source: BMC Pulm Med. 2023 Nov 21;23:459. doi: 10.1186/s12890-023-02720-0 (PMC10664596; doi:10.1186/s12890-023-02720-0)
Supplement: Supplementary file 1 — Additional file 1: Supplementary Figure S1. Scatter plot of the association of hand grip strength with pulmonary function. Supplementary Figure S2. Forest plot of the association of hand grip strength with pulmonary function. Supplementary Figure S3. Leave-one-out sensitivity analysis of the association of hand grip strength with pulmonary function. Supplementary Figure S4. Funnel plot of the association of hand grip strength with pulmonary function. Supplementary Table S1. Baseline characteristics of hand grip strength and pulmonary function. Supplementary Table S2. Single nucleotide polymorphisms used as instrumental variables in the Mendelian randomization analysis of right-hand grip strength. Supplementary Table S3. Single nucleotide polymorphisms used as instrumental variables in the Mendelian randomization analysis of left-hand grip strength. Supplementary Table S4. SNPs of RHGS excluded from Mendelian randomization analysis. Supplementary Table S5. SNPs of LHGS excluded from Mendelian randomization analysis. STROBE-MR checklist of recommended items to address in reports of Mendelian randomization studies. [file 12890_2023_2720_MOESM1_ESM.zip › sumplmentary/Supplementary Tables.pdf]

Supplementary Table S1: Baseline characteristics of hand grip strength and pulmonary function.

Exposures/Outcomes	Trait	GWAS ID	Year	Consortium	Population	Sample size	Author	*n* SNPs
HGS	RHGS	ukb-b-10215	2018	MRC-IEU	European	461,089	Ben Elsworth	9,851,867
	LHGS	ukb-b-7478	2018	MRC-IEU	European	461,026	Ben Elsworth	9,851,867
PF	FVC	ukb-b-7953	2018	MRC-IEU	European	421,986	Ben Elsworth	9,851,867
	FEV1	ukb-b-19657	2018	MRC-IEU	European	421,986	Ben Elsworth	9,851,867
	FEV1/FVC	ebi-a-GCST007431	2019	NA	European	321,047	Shrine N	19,671,887

HGS, handgrip strength, RHGS, right handgrip strength; LHGS, left handgrip strength; PF, pulmonary function; FVC, forced vital capacity; FEV1, forced expiratory volume in the first second. NA, not available.

Supplementary Table S2: Single nucleotide polymorphisms used as instrumental variables in the Mendelian randomization analysis of right-hand grip strength.

SNP	Chr	EA	NEA	Beta	SE	*p*-Value	R^2	F-Value
rs10798876	1	G	C	0.009	0.001	4.30E-09	7.48E-05	34
rs4927015	1	A	G	0.013	0.002	5.00E-18	1.62E-04	75
rs35304341	1	A	G	-0.014	0.003	4.00E-08	6.53E-05	30
rs56144131	1	C	T	-0.013	0.002	3.80E-10	8.51E-05	39
rs7549184	1	A	G	0.011	0.002	5.80E-09	7.35E-05	34
rs12562146	1	A	T	0.012	0.002	2.00E-08	6.83E-05	31
rs2147461	1	C	T	0.013	0.002	6.10E-09	7.33E-05	34
rs6693567	1	T	C	-0.010	0.002	6.30E-09	7.32E-05	34
rs150330307	1	C	T	-0.033	0.004	1.20E-14	1.29E-04	60
rs1442883	2	A	C	-0.011	0.002	5.80E-10	8.32E-05	38
rs35833641	2	G	A	0.009	0.002	8.50E-09	7.19E-05	33
rs2894602	2	G	A	0.010	0.002	9.90E-09	7.13E-05	33
rs7565148	2	G	T	-0.010	0.001	3.60E-12	1.05E-04	48
rs7576964	2	T	G	0.010	0.002	5.30E-10	8.37E-05	39
rs34030812	2	C	T	-0.009	0.002	3.30E-09	7.59E-05	35
rs7575451	2	G	C	-0.011	0.002	9.30E-12	1.01E-04	46
rs12616285	2	G	T	0.012	0.002	5.40E-09	7.39E-05	34
rs6715064	2	T	C	-0.009	0.002	9.60E-09	7.14E-05	33
rs1641457	2	G	T	0.013	0.002	2.40E-13	1.16E-04	54
rs3771498	2	T	C	0.014	0.001	1.90E-21	1.96E-04	90
rs12052508	2	T	C	-0.013	0.002	4.70E-09	7.44E-05	34
rs10193039	2	T	A	-0.010	0.002	4.10E-10	8.47E-05	39
rs2194747	2	G	A	0.010	0.002	1.90E-09	7.83E-05	36
rs6792762	3	A	G	-0.009	0.002	1.80E-09	7.85E-05	36
rs1440152	3	G	C	0.008	0.001	3.50E-08	6.59E-05	30
rs35701422	3	C	T	-0.009	0.002	1.50E-08	6.95E-05	32
rs35457492	3	C	A	0.008	0.001	2.20E-08	6.78E-05	31
rs9757079	3	T	C	0.010	0.002	8.40E-10	8.17E-05	38
rs71298370	3	A	G	0.017	0.003	8.90E-10	8.15E-05	38
rs34587452	4	C	G	-0.011	0.002	9.10E-10	8.13E-05	38
rs7657558	4	G	T	0.011	0.002	1.10E-10	9.03E-05	42
rs114924396	4	G	A	-0.019	0.003	8.20E-09	7.21E-05	33
rs997850	4	C	G	-0.009	0.002	3.70E-09	7.54E-05	35
rs13169333	5	C	T	0.009	0.002	4.30E-08	6.51E-05	30
rs75457267	5	T	C	-0.019	0.003	3.40E-08	6.60E-05	30
rs12522139	5	G	T	-0.011	0.002	6.80E-09	7.29E-05	34
rs6882168	5	T	C	-0.009	0.002	7.90E-09	7.22E-05	33
rs13356200	5	G	T	-0.009	0.002	1.80E-09	7.85E-05	36
rs6870324	5	G	C	-0.010	0.002	3.00E-09	7.63E-05	35
rs13355365	5	T	C	-0.008	0.002	3.40E-08	6.61E-05	30
rs9388051	6	A	G	0.011	0.002	2.70E-08	6.70E-05	31
rs113835839	6	T	C	-0.010	0.002	1.10E-08	7.09E-05	33

rs645144	6	C	T	-0.009	0.002	4.30E-08	6.51E-05	30
rs721101	6	C	T	0.009	0.002	1.60E-08	6.93E-05	32
rs77485342	6	T	C	0.035	0.006	2.60E-10	8.67E-05	40
rs1125	6	A	G	-0.010	0.002	1.90E-10	8.80E-05	41
rs9396861	6	A	C	-0.010	0.002	5.70E-10	8.33E-05	38
rs35175534	6	C	A	-0.019	0.002	4.20E-16	1.43E-04	66
rs852520	7	A	C	-0.009	0.002	1.50E-08	6.95E-05	32
rs6962338	7	G	A	-0.020	0.004	2.00E-08	6.84E-05	32
rs2389763	7	C	T	-0.008	0.002	3.80E-08	6.56E-05	30
rs2717351	7	G	A	0.013	0.002	3.40E-12	1.05E-04	48
rs9639938	7	G	C	0.009	0.001	5.20E-09	7.40E-05	34
rs4730984	7	T	G	0.010	0.002	1.80E-09	7.84E-05	36
rs4737446	8	T	G	0.010	0.002	2.40E-10	8.70E-05	40
rs62509875	8	G	A	-0.013	0.002	2.40E-11	9.67E-05	45
rs1486925	8	C	T	-0.010	0.002	2.90E-09	7.65E-05	35
rs116922558	9	G	A	-0.025	0.004	1.80E-10	8.83E-05	41
rs7034200	9	A	C	0.009	0.001	5.30E-09	7.39E-05	34
rs11998884	9	T	C	0.017	0.003	2.80E-08	6.68E-05	31
rs10761411	9	T	C	-0.011	0.002	1.30E-08	7.02E-05	32
rs72820369	10	T	A	0.016	0.002	4.40E-12	1.04E-04	48
rs4751671	10	A	G	0.008	0.002	4.10E-08	6.53E-05	30
rs12412806	10	A	G	-0.009	0.002	3.00E-08	6.66E-05	31
rs2273555	10	A	G	0.011	0.002	4.20E-13	1.14E-04	53
rs4962700	10	G	C	0.009	0.002	1.20E-08	7.04E-05	32
rs1556659	10	T	C	0.018	0.002	3.80E-30	2.82E-04	130
rs11022513	11	T	C	-0.009	0.002	1.10E-09	8.07E-05	37
rs2244621	11	T	C	0.012	0.002	4.50E-08	6.49E-05	30
rs61389091	11	T	C	0.022	0.004	3.60E-09	7.55E-05	35
rs34845616	11	A	G	0.010	0.002	1.70E-08	6.90E-05	32
rs12790261	11	A	C	-0.026	0.003	2.00E-22	2.06E-04	95
rs72977282	11	A	T	-0.017	0.002	1.20E-28	2.67E-04	123
rs6592737	11	T	A	-0.009	0.002	2.00E-09	7.81E-05	36
rs1635527	12	C	G	0.010	0.001	6.80E-11	9.23E-05	43
rs7301953	12	A	G	-0.012	0.002	6.40E-13	1.12E-04	52
rs10846071	12	T	C	-0.016	0.002	6.50E-25	2.30E-04	106
rs12823922	12	G	A	-0.011	0.002	1.60E-10	8.86E-05	41
rs7963801	12	C	T	-0.011	0.002	8.40E-14	1.21E-04	56
rs12316046	12	G	A	-0.016	0.002	3.80E-26	2.43E-04	112
rs7953280	12	C	G	-0.009	0.001	1.80E-09	7.84E-05	36
rs2296316	14	C	T	-0.008	0.002	4.60E-08	6.48E-05	30
rs12889267	14	G	A	-0.012	0.002	6.70E-10	8.26E-05	38
rs935728	14	T	C	0.010	0.002	1.80E-09	7.86E-05	36
rs7148603	14	A	G	0.009	0.002	4.70E-09	7.44E-05	34
rs9652468	15	A	G	-0.013	0.002	3.30E-13	1.15E-04	53
rs4553566	15	C	T	-0.009	0.001	4.60E-10	8.43E-05	39
rs12914702	15	A	G	0.011	0.002	1.90E-10	8.79E-05	41
rs12899474	15	T	C	-0.015	0.002	5.40E-10	8.36E-05	39
rs11642954	16	A	G	-0.013	0.002	2.20E-12	1.07E-04	49
rs8055199	16	A	G	-0.009	0.002	1.20E-08	7.04E-05	32
rs7206195	16	T	C	-0.015	0.002	2.80E-15	1.35E-04	62
rs62037412	16	A	G	0.009	0.002	1.90E-09	7.81E-05	36
rs3848369	16	T	C	-0.010	0.002	4.70E-10	8.41E-05	39
rs76749769	16	T	C	0.014	0.003	2.20E-08	6.79E-05	31
rs4784329	16	C	A	-0.013	0.002	9.00E-19	1.70E-04	78
rs7214252	17	A	G	-0.010	0.002	2.00E-08	6.83E-05	31
rs2587505	17	C	T	-0.009	0.002	8.10E-10	8.19E-05	38
rs4793658	17	C	A	-0.014	0.002	3.80E-08	6.56E-05	30
rs56074046	17	A	G	-0.009	0.002	4.40E-09	7.47E-05	34
rs635538	18	A	G	-0.022	0.003	1.50E-16	1.48E-04	68
rs4369779	18	C	T	0.017	0.002	3.40E-21	1.94E-04	89
rs34217742	19	A	T	0.015	0.002	1.40E-10	8.94E-05	41
rs7249	19	T	C	0.008	0.002	4.00E-08	6.54E-05	30
rs4802848	19	C	G	0.011	0.002	3.80E-11	9.48E-05	44
rs79723785	19	C	T	-0.034	0.006	1.60E-08	6.92E-05	32
rs911642	20	T	C	0.009	0.002	2.00E-08	6.83E-05	31

rs6063504	20	G	C	0.009	0.001	7.80E-09	7.23E-05	33
rs2226685	21	C	T	0.010	0.002	3.10E-09	7.62E-05	35
rs6006984	22	C	T	0.010	0.002	5.10E-10	8.38E-05	39

Supplementary Table S3: Single nucleotide polymorphisms used as instrumental variables in the Mendelian randomization analysis of left-hand grip strength.

SNP	Chr	EA	NEA	Beta	SE	*p*-Value	R^2	F-Value
rs6680160	1	G	A	0.010	0.002	6.00E-11	9.29E-05	43
rs7516571	1	G	A	0.009	0.002	3.10E-08	6.65E-05	31
rs150330307	1	C	T	-0.031	0.004	2.90E-13	1.16E-04	53
rs2800789	1	C	A	0.008	0.001	3.10E-08	6.64E-05	31
rs10788958	1	G	C	0.014	0.002	1.00E-19	1.79E-04	83
rs4335354	1	A	C	-0.009	0.002	4.60E-09	7.45E-05	34
rs1884447	1	A	G	0.008	0.002	2.30E-08	6.77E-05	31
rs61818100	1	C	T	0.013	0.002	6.20E-09	7.32E-05	34
rs6433478	2	C	T	0.009	0.001	1.50E-09	7.91E-05	36
rs12473732	2	T	C	0.011	0.001	1.40E-13	1.19E-04	55
rs7571789	2	C	T	0.013	0.001	3.00E-18	1.65E-04	76
rs7575451	2	G	C	-0.010	0.002	3.80E-10	8.51E-05	39
rs1434095	2	C	T	0.014	0.002	5.30E-10	8.37E-05	39
rs17630248	2	C	T	-0.009	0.002	4.10E-09	7.50E-05	35
rs1981612	2	A	C	0.009	0.002	1.00E-09	8.09E-05	37
rs1641457	2	G	T	0.012	0.002	1.40E-11	9.90E-05	46
rs10176878	2	C	T	-0.013	0.002	8.60E-12	1.01E-04	47
rs61286123	2	C	T	-0.010	0.002	1.20E-08	7.06E-05	33
rs34030812	2	C	T	-0.010	0.002	4.10E-11	9.45E-05	44
rs10205394	2	C	G	-0.011	0.002	1.10E-09	8.06E-05	37
rs1551042	3	C	A	-0.011	0.002	7.40E-13	1.12E-04	51
rs9866627	3	A	C	-0.016	0.003	5.70E-09	7.36E-05	34
rs112485536	3	T	C	0.016	0.003	9.40E-09	7.15E-05	33
rs62253653	3	G	A	0.011	0.002	5.50E-11	9.33E-05	43
rs6802071	3	T	C	-0.009	0.002	3.80E-10	8.50E-05	39
rs71298370	3	A	G	0.015	0.003	3.80E-08	6.56E-05	30
rs13091492	3	G	A	-0.008	0.002	3.30E-08	6.63E-05	31
rs4677601	3	G	A	0.009	0.001	1.10E-09	8.07E-05	37
rs35609019	4	C	G	0.009	0.002	7.70E-10	8.21E-05	38
rs13107325	4	T	C	-0.026	0.003	1.80E-20	1.86E-04	86
rs56338231	4	G	A	-0.011	0.002	1.70E-10	8.86E-05	41
rs997850	4	C	G	-0.009	0.002	6.30E-09	7.32E-05	34
rs34722008	4	A	G	0.009	0.002	3.30E-08	6.62E-05	31
rs2850379	4	A	C	-0.008	0.002	3.30E-08	6.62E-05	31
rs75497896	4	C	T	-0.021	0.003	9.10E-10	8.14E-05	38
rs116409670	5	T	C	-0.015	0.003	2.60E-08	6.72E-05	31
rs13356200	5	G	T	-0.009	0.002	9.70E-09	7.14E-05	33
rs6882168	5	T	C	-0.009	0.002	2.80E-09	7.66E-05	35
rs113918482	5	G	A	-0.010	0.002	1.50E-08	6.94E-05	32
rs2974438	5	A	G	-0.010	0.002	3.30E-08	6.62E-05	31
rs9371201	6	T	C	-0.009	0.002	3.00E-09	7.64E-05	35
rs77485342	6	T	C	0.033	0.006	3.50E-09	7.57E-05	35
rs35175534	6	C	A	-0.016	0.002	3.60E-12	1.05E-04	48
rs2038760	6	T	C	-0.012	0.002	6.60E-09	7.30E-05	34
rs9371881	6	A	G	0.009	0.002	9.60E-10	8.11E-05	37
rs4621706	7	T	C	-0.012	0.002	6.00E-15	1.32E-04	61
rs12533765	7	G	A	-0.009	0.002	2.60E-08	6.73E-05	31
rs16870531	7	T	C	0.011	0.002	1.30E-10	8.96E-05	41
rs12673062	7	A	G	-0.011	0.002	2.60E-09	7.69E-05	35
rs73307079	7	C	T	0.011	0.002	1.40E-09	7.95E-05	37
rs6962338	7	G	A	-0.021	0.004	3.20E-09	7.60E-05	35
rs17282763	7	C	T	0.009	0.002	4.40E-08	6.50E-05	30
rs821100	8	G	A	-0.010	0.002	1.50E-09	7.93E-05	37
rs59116179	8	T	C	0.009	0.002	2.40E-08	6.75E-05	31
rs4737446	8	T	G	0.010	0.002	1.20E-10	8.98E-05	41
rs1486925	8	C	T	-0.010	0.002	6.30E-11	9.27E-05	43

rs10097417	8	G	A	-0.013	0.002	1.90E-11	9.78E-05	45
rs7026798	9	C	T	0.008	0.002	4.60E-08	6.48E-05	30
rs116922558	9	G	A	-0.022	0.004	2.10E-08	6.80E-05	31
rs2789514	9	A	G	0.012	0.002	4.90E-08	6.46E-05	30
rs10988217	9	G	A	-0.009	0.002	1.70E-09	7.86E-05	36
rs35236379	10	T	G	0.012	0.002	5.80E-09	7.35E-05	34
rs4962700	10	G	C	0.009	0.002	3.20E-08	6.64E-05	31
rs772014	10	G	A	-0.011	0.002	2.70E-12	1.06E-04	49
rs10821939	10	A	G	-0.009	0.002	4.90E-10	8.40E-05	39
rs1556659	10	T	C	0.016	0.002	2.50E-26	2.44E-04	113
rs72977282	11	A	T	-0.016	0.002	7.80E-25	2.30E-04	106
rs10831903	11	T	C	0.009	0.002	8.10E-10	8.19E-05	38
rs61389091	11	T	C	0.026	0.004	2.40E-12	1.07E-04	49
rs34845616	11	A	G	0.011	0.002	4.00E-10	8.48E-05	39
rs10846071	12	T	C	-0.017	0.002	5.30E-28	2.61E-04	120
rs11168357	12	A	G	-0.010	0.002	2.50E-08	6.75E-05	31
rs4575361	12	T	A	-0.011	0.002	1.60E-11	9.86E-05	45
rs12316046	12	G	A	-0.017	0.002	5.00E-30	2.81E-04	130
rs7963801	12	C	T	-0.010	0.002	4.60E-12	1.04E-04	48
rs56060323	13	T	C	0.009	0.002	1.50E-08	6.95E-05	32
rs12889267	14	G	A	-0.014	0.002	4.70E-12	1.04E-04	48
rs7148603	14	A	G	0.010	0.002	1.60E-09	7.90E-05	36
rs2359239	14	T	C	-0.009	0.002	6.90E-09	7.28E-05	34
rs10144445	14	G	C	-0.009	0.002	1.90E-09	7.83E-05	36
rs12906830	15	C	T	0.011	0.002	9.30E-13	1.11E-04	51
rs17466480	15	G	A	-0.012	0.002	9.80E-15	1.30E-04	60
rs12914702	15	A	G	0.011	0.002	9.30E-11	9.10E-05	42
rs7176095	15	G	A	-0.013	0.002	1.70E-09	7.87E-05	36
rs13337177	16	T	G	-0.014	0.002	1.60E-13	1.18E-04	54
rs11642954	16	A	G	-0.012	0.002	4.50E-10	8.43E-05	39
rs217181	16	T	C	0.012	0.002	2.10E-10	8.76E-05	40
rs9944324	16	G	A	-0.009	0.002	1.20E-08	7.04E-05	32
rs7197751	16	T	G	-0.009	0.002	1.40E-09	7.95E-05	37
rs3814877	16	T	G	0.011	0.002	3.40E-12	1.05E-04	48
rs11076004	16	A	G	-0.012	0.002	2.10E-14	1.27E-04	58
rs999493	17	A	G	0.013	0.002	6.20E-17	1.52E-04	70
rs2587505	17	C	T	-0.009	0.002	2.30E-09	7.74E-05	36
rs635538	18	A	G	-0.022	0.003	3.80E-16	1.44E-04	66
rs4308051	18	G	T	0.016	0.002	1.40E-18	1.68E-04	77
rs143002906	18	T	C	0.026	0.005	9.80E-09	7.13E-05	33
rs10403906	19	A	G	-0.010	0.001	1.50E-11	9.88E-05	46
rs11669079	19	T	A	0.011	0.002	1.90E-11	9.78E-05	45
rs8101782	19	C	A	0.010	0.002	2.90E-08	6.67E-05	31
rs8108461	19	C	T	0.010	0.002	2.60E-10	8.67E-05	40
rs143384	20	G	A	0.021	0.002	1.50E-43	4.15E-04	192
rs9611273	22	T	C	0.011	0.002	4.50E-10	8.43E-05	39
rs6006984	22	C	T	0.010	0.002	2.60E-09	7.69E-05	35

Chr: chromosome; EA: effect allele; NEA: non-effect allele; SE: standard error; SNP: single-nucleotide polymorphisms

Supplementary Table S4: SNPs of RHGS excluded from Mendelian randomization analysis.

Exposure	SNP ID	PhenoScanner traits
RHGS	rs58670122,rs10798483,rs6693965,rs1952256,rs823130,rs10799428, rs4121165,rs1892425,rs1550115,rs6711390,rs1840753,rs1047891, rs7652177,rs2194411,rs2362972,rs62234790,rs2341184,rs9853018, rs13146142,rs13106087,rs13107325,rs4868110,rs2431112,rs2631360, rs2322754,rs9322822,rs7451021,rs11243202,rs9267806,rs185320691,rs113315602,rs1885690,rs4549685,rs112330055,rs7790322,rs6977081, rs10278546,rs6473015,rs7871404,rs2208562,rs113851275,rs600038, rs4752689, rs12763284, rs11039348, rs10770125, rs76895963, rs10784502, rs4768725,rs3118914,rs10483727,rs2871865,rs2165241,rs1210479,rs246181,rs7196917,rs248831,rs478557	FVC/FEV1/Asthma /Peak expiratory flow/ Pulmonary Embolism/ Bronchitis/ Emphysema

	4rs2854152,rs1043515,rs12452505,rs56365901,rs10520770,rs36065733,rs7266065,rs143384

Supplementary Table S5: SNPs of LHGS excluded from Mendelian randomization analysis.

Exposure	SNP ID	PhenoScanner traits
LHGS	rs1044299,rs11121542,rs4121165,rs58670122,rs823130,rs11204664, rs6689375,rs11125803,rs3819121,rs10934857,rs4498020,rs2871960, rs13146142,rs34587452,rs13106087,rs55681913,rs2431112,rs2631360rs185320691,rs12528131,rs9388769,rs113315602,rs723588,rs11243202,rs4713506,rs41271299,rs11769549,rs13227429, rs6977081, rs4398863, rs4739739, rs7856625, rs16910750, rs11002322, rs10786706, rs11003014,rs4930236, rs12790261, rs7124681, rs76895963, rs7970350, rs11111267, rs3118903, rs28542042, rs3959716,rs2871865, rs7196917, rs181766, rs113434679, rs755547, rs2532111, rs62081464, rs35054365, rs4811040	FVC/FEV1/Asthma /Peak expiratory flow/ Long-standing illness, disability or infirmity
