# Supplementary figures and images for: Causal associations between hand grip strength and pulmonary function: a two-sample Mendelian randomization study
Source: BMC Pulm Med. 2023 Nov 21;23:459. doi: 10.1186/s12890-023-02720-0 (PMC10664596; doi:10.1186/s12890-023-02720-0)

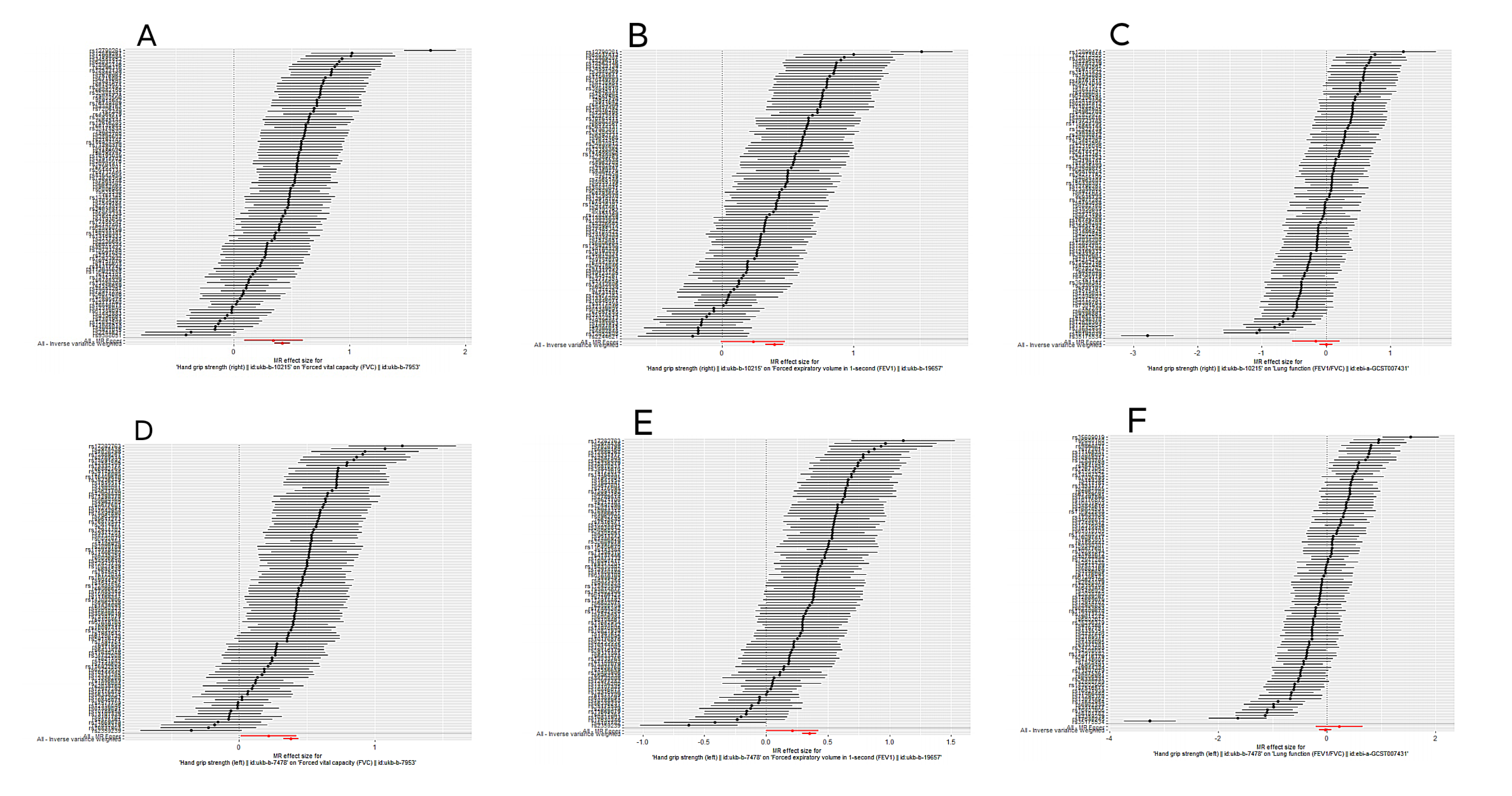

Supplement: Supplementary file 1 — Additional file 1: Supplementary Figure S1. Scatter plot of the association of hand grip strength with pulmonary function. Supplementary Figure S2. Forest plot of the association of hand grip strength with pulmonary function. Supplementary Figure S3. Leave-one-out sensitivity analysis of the association of hand grip strength with pulmonary function. Supplementary Figure S4. Funnel plot of the association of hand grip strength with pulmonary function. Supplementary Table S1. Baseline characteristics of hand grip strength and pulmonary function. Supplementary Table S2. Single nucleotide polymorphisms used as instrumental variables in the Mendelian randomization analysis of right-hand grip strength. Supplementary Table S3. Single nucleotide polymorphisms used as instrumental variables in the Mendelian randomization analysis of left-hand grip strength. Supplementary Table S4. SNPs of RHGS excluded from Mendelian randomization analysis. Supplementary Table S5. SNPs of LHGS excluded from Mendelian randomization analysis. STROBE-MR checklist of recommended items to address in reports of Mendelian randomization studies. [file 12890_2023_2720_MOESM1_ESM.zip › sumplmentary/Supplementary Figure S2.tif]

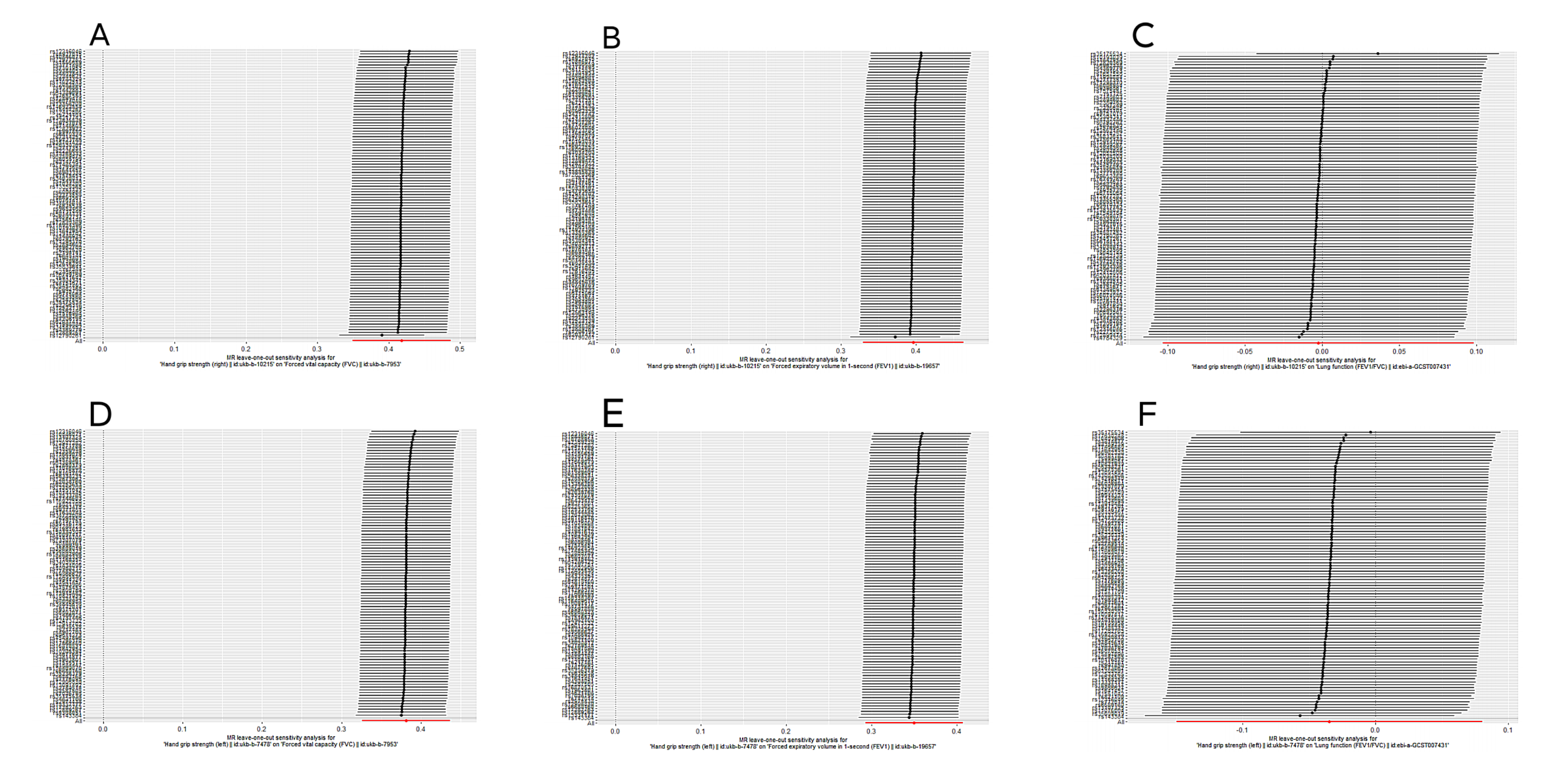

Supplement: Supplementary file 1 — Additional file 1: Supplementary Figure S1. Scatter plot of the association of hand grip strength with pulmonary function. Supplementary Figure S2. Forest plot of the association of hand grip strength with pulmonary function. Supplementary Figure S3. Leave-one-out sensitivity analysis of the association of hand grip strength with pulmonary function. Supplementary Figure S4. Funnel plot of the association of hand grip strength with pulmonary function. Supplementary Table S1. Baseline characteristics of hand grip strength and pulmonary function. Supplementary Table S2. Single nucleotide polymorphisms used as instrumental variables in the Mendelian randomization analysis of right-hand grip strength. Supplementary Table S3. Single nucleotide polymorphisms used as instrumental variables in the Mendelian randomization analysis of left-hand grip strength. Supplementary Table S4. SNPs of RHGS excluded from Mendelian randomization analysis. Supplementary Table S5. SNPs of LHGS excluded from Mendelian randomization analysis. STROBE-MR checklist of recommended items to address in reports of Mendelian randomization studies. [file 12890_2023_2720_MOESM1_ESM.zip › sumplmentary/Supplementary Figure S3.tif]
